# In vivo evaluation of the enamel wear of primary molar against four types of crowns using the intra-oral scanner

**DOI:** 10.1186/s12903-024-05206-5

**Published:** 2024-11-27

**Authors:** Diana Mohamed Amer, Abeer Mostafa Abdellatif

**Affiliations:** Pediatric Dentistry Department, Faculty of Dentistry, Mansoura University, Mansoura, Egypt

**Keywords:** Wear, Primary molar, NuSmile, CAD/CAM, Zirconia, Hybrid, Stainless steel, Crown, Intraoral scanner

## Abstract

**Background:**

Primary tooth wear is a common phenomenon that affects chewing ability, dental sensitivity, aesthetics, and occlusion. This study was conducted to compare the antagonistic enamel wear of primary molars opposed to four different crown materials.

**Methods:**

Forty lower second primary molars of children aged 4–8 years were allocated into 4 groups: Group 1 (*n* = 10): received stainless steel crowns; Group 2 (*n* = 10): received prefabricated commercially available zirconia crowns (NuSmile^®^); Group 3 (*n* = 10): received locally manufactured zirconia crowns created via the CAD/CAM system; and Group 4 (*n* = 10): received locally manufactured hybrid ceramic (Vita Enamic^®^) crowns created via the CAD/CAM system. All the crowns were cemented with resin-modified glass ionomer cement. The upper arch was scanned with a 3D intraoral scanner immediately (baseline), 6 months, and 1 year after crown cementation to evaluate the wear of the natural enamel of the antagonistic primary molar. The resultant scans were compared via Exocad software to measure the amount of linear wear of the mesiopalatal cusp of the primary upper second molar.

**Results:**

The analysed data revealed statistically significant differences between the studied groups. The highest mean wear was detected in the CAD/CAM zirconia crown group, followed by the NuSmile zirconia crown group and then the CAD/CAM hybrid crown group, and the lowest mean wear was detected for the stainless-steel crown group.

**Conclusion:**

Compared with stainless steel crowns, aesthetic crowns cause more wear in the antagonistic primary molar enamel. CAD/CAM zirconia crowns induce the greatest amount of wear, followed by NuSmile zirconia crowns. The CAD/CAM hybrid crown is an aesthetic tooth-coloured crown that causes less wear of the opposing enamel than zirconia crowns do.

**Trial registration:**

The ClinicalTrials.gov Protocol Registration and Results System (PRS) has this RCT registered as (NCT06456879) on 07/06/2024.

**Supplementary Information:**

The online version contains supplementary material available at 10.1186/s12903-024-05206-5.

## Background

Wear in primary teeth is a common phenomenon caused by the loss of enamel and dentin from occlusal surfaces. Ideally, dental restorative materials should not accelerate the wear of the opposing enamel surface [1]. An abnormal loss of tooth structure can affect chewing ability, dental sensitivity, aesthetics, and occlusion [2]. Primary and permanent teeth have different levels of abrasiveness due to variations in their morphologies, such as enamel and dentine thicknesses [3], physical characteristics [4], and biting forces of children and adults [5].

Crowns are the best definitive restorations for primary teeth because of their high sealing ability [6]. Stainless steel crowns (SSCs) have many advantages, as they are functional, durable and cost effective; however, they are the least attractive to children or their parents because of their silver metal colour [7]. Aesthetic dentistry is currently an essential component of dental practice. Zirconia crowns are highly aesthetic, biocompatible, durable and functional [8, 9]. However, the high cost and abrasiveness of zirconia can be considered significant drawbacks [10–12]. Commercially available prefabricated zirconia crowns include Cheng Crowns^®^, EZ Pedo^®^, Kinder^®^, and NuSmile^®^.

Currently, the most active area in the dental industry is the development of new computer-aided design/computer-aided manufacturing (CAD/CAM) materials [13, 14]. Locally manufactured zirconia crowns via CAD/CAM technology offer a satisfactory cost-effective aesthetic restorative option [15]. Ceramics account for the majority of CAD/CAM materials, but significant advancements have been made in CAD/CAM hybrid ceramic materials, which combine the advantages of ceramics and resin-based materials.

Compared with ceramics, hybrid materials have many advantages, as their modulus of elasticity is very similar to that of dentin, and they can be more easily fabricated and repaired [16]. However, hybrid materials are inferior to ceramics in other aspects, such as mechanical properties, biocompatibility, and material loss [17, 18]. Vita Enamic is a representative example of a hybrid material. Vita Enamic (ENA) is a polymer-infiltrated ceramic, as its dominant ceramic is reinforced by a network of polymers. It consists of an interpenetrating network of appropriately incorporated ceramic and composite resins [19].

Michou et al. (2020) [20] reported that an intraoral scanner supported by specific software showed good performance for the early detection and monitoring of tooth wear in vitro and has promising potential for in vivo application. Additionally, Prabhu et al. 2022 [21] evaluated and compared the clinical wear of prefabricated stainless steel crowns and CAD/CAM zirconia crowns (custom-made) in primary molars. An intraoral scanner was used to scan the lower arch of the tooth to be crowned and the upper arch. The researchers reported that SSCs resulted in less wear of the opposing tooth enamel but that a tooth-coloured crown was preferred by parents and patients as a treatment option.

Therefore, a question of special interest concerns the behaviour of wear in primary tooth enamel in comparison to various crown materials, particularly in molar regions where the masticatory force is high. To the best of our knowledge, the enamel wear of primary teeth has been investigated in many in vitro studies [22–27], but few in vivo studies exist [21].

### Study aim

The objective of this study was to compare the antagonistic enamel wear of primary molars to that of four different crown materials: stainless steel crowns, prefabricated commercially available zirconia crowns (NuSmile^®^) (NZCs), locally manufactured zirconia crowns created via CAD/CAM technology (CCZCs), and locally manufactured hybrid ceramic crowns created via CAD/CAM technology (CCHCs).

## Methods

### Study design

This study was a randomized controlled clinical trial, and followed the recommendations of the CONSORT guidelines. ClinicalTrials.gov Identifier: NCT06456879. The sample size was estimated on the basis of the mean wear between different types of full coronal coverage retrieved from previous research [22]. The sample size was calculated via the G power program version 3.1.9.7. The total calculated sample size was 9 patients in each group. Children were selected from the Faculty of Dentistry (the Paediatric Dental Clinic), Mansoura University. Forty lower second primary molars were allocated randomly into 4 groups: Group 1 (*n* = 10), which received 3 M™ ESPE™ SSCs for primary molars; Group 2 (*n* = 10), which received commercially available prefabricated zirconia crowns (NuSmile^®^) (NZC); Group 3 (*n* = 10), which received zirconia crowns locally manufactured via CAD/CAM technology (CCZC); and Group 4 (*n* = 10), which received hybrid ceramic (Vita Enamic^®^) crowns locally manufactured via CAD/CAM technology (CCHC).

### Eligibility criteria

The eligibility criteria were as follows: 4–8-year-old children with definitely positive or positive behaviour according to the Frankl behaviour rating scale; lower second primary molar indicated for crown restoration; soundness of the opposing upper second primary molar; and the absence of periodontal disease, occlusal problems or habits as bruxism.

### Ethics approval and consent to participate

Ethical approval for all study protocol steps was obtained from the Mansoura Research Ethics Committee, Faculty of Dentistry, Mansoura University, with reference number M01010222. Written informed consent was provided by the parents prior to the examination and treatment of their children.

### Method of randomization

Simple randomization was performed via the randomization formula in Excel (Microsoft, Wash, USA). Random numbers were printed on sheets of paper, which were then folded; next, all the papers were collected in a box to ensure concealment of the allocation. Each child was allowed to choose a paper from the box and then allocated to the matching group.

### The laboratory manufacture of CAD/CAM crowns

CAD/CAM crowns were designed in a dental technical laboratory. The zirconia crowns (AMANN GIRRBACH Ceramill Zolid HT) were milled in dry processing mode, whereas the hybrid ceramic crowns (Vita Enamic^®^) were milled in wet processing mode in a CAD-CAM milling machine (Amann Girrbach Ceramill Motion 2) to produce different sizes of prefabricated crowns for the second primary molars.

NuSmile^®^ crowns were used to design the locally manufactured CAD/CAM crowns to produce different crown sizes. Using an Open Technologies Optical 3D Scanner, the inner and outer faces of the NuSmile crown were scanned after being sprayed with the digital scanner’s spray marker [9]. CAD/CAM crowns were designed with the same inner and outer surfaces and the same thickness of the NuSmile crowns.

**Fig. 1:**
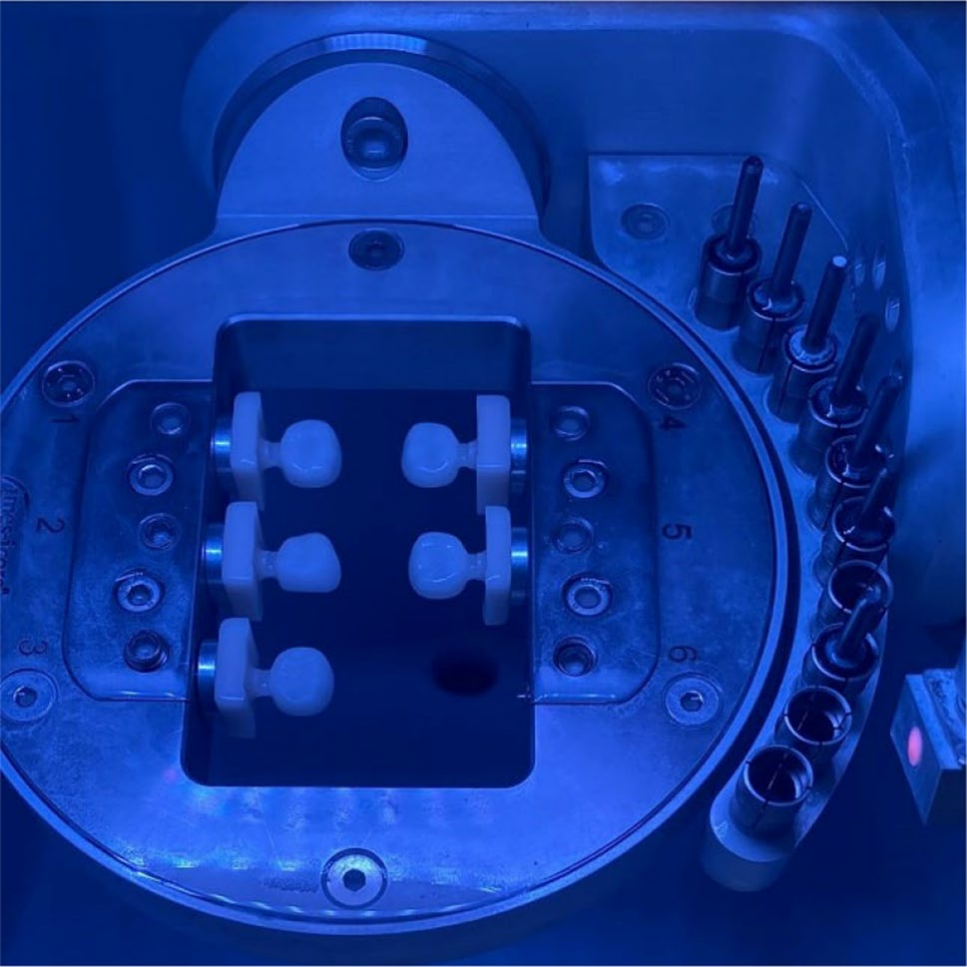
Milling process of the CAD/CAM hybrid crowns

**Fig. 2:**
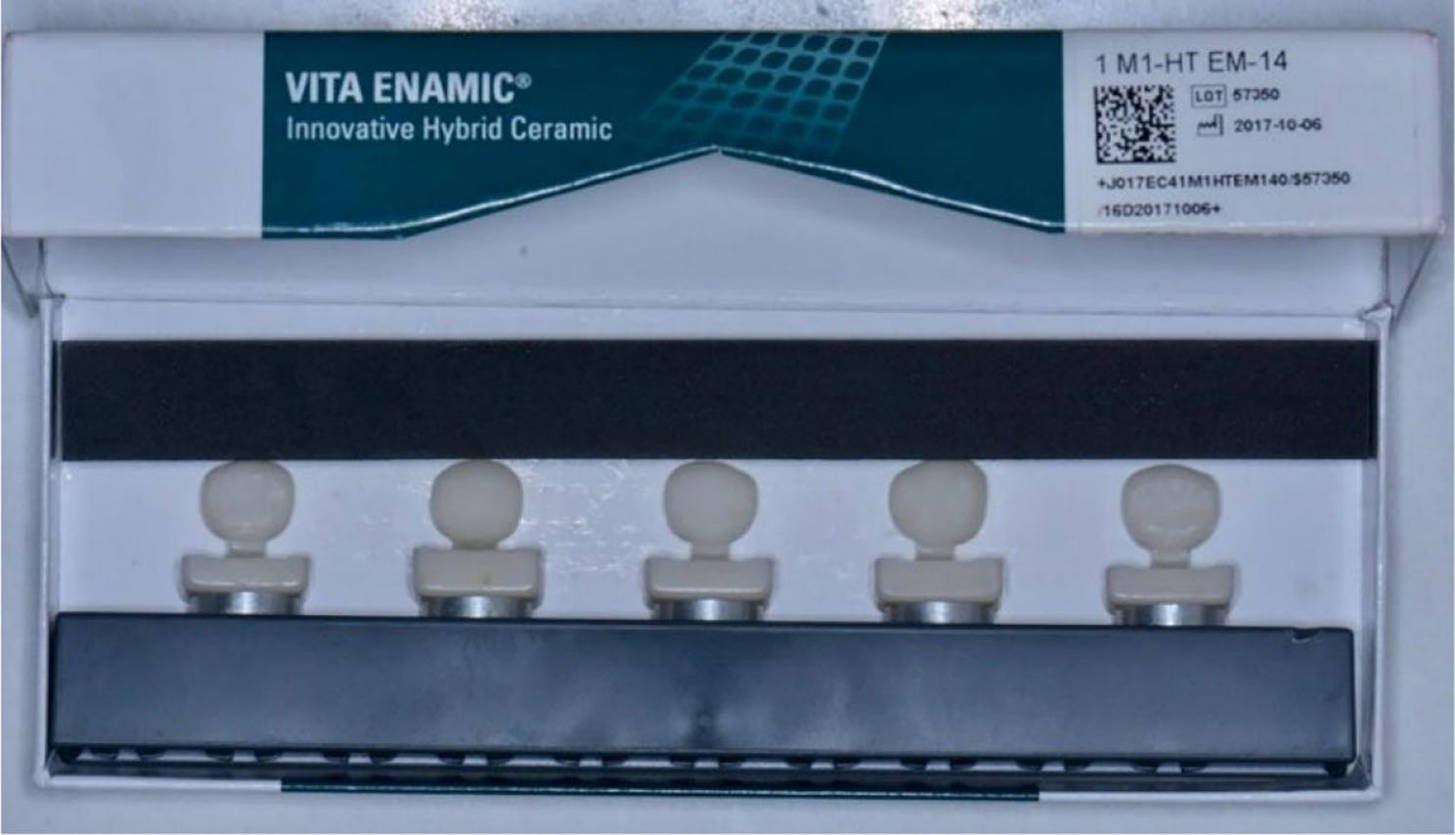
VITA ENAMIC hybrid ceramic blocks after milling

**Fig. 3:**
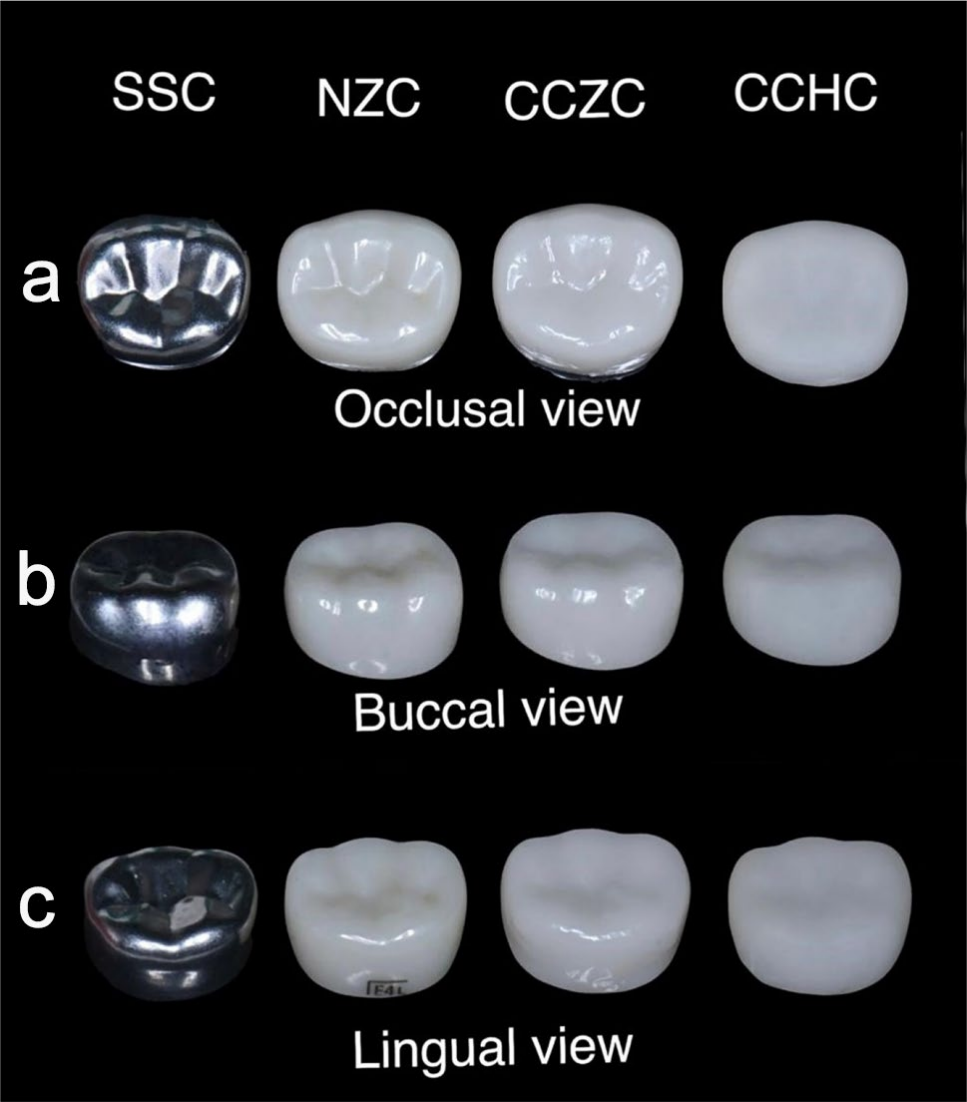
The four types of crowns from different aspects. (**A**) The four types of crowns from the occlusal aspect. (**B**) The four types of crowns from the buccal aspect. (**C**) The four types of crowns from the lingual aspect

For fabrication, the CAM device received an STL file. Each milled crown was separated from the CAD/CAM blocks. The finishing and polishing procedures were performed by a dental technician according to the manufacturer’s specifications. This procedure was repeated for every crown size. Finally, the crowns were encoded in various sizes and then collected in a special box with separators to produce a complete set of prefabricated crowns that had been locally manufactured via CAD/CAM technology (Figures 1, 2, 3).

### Clinical work

Clinical and radiographic examinations were performed to ensure that the primary molar could be included in the study. Both the child and his or her parents received oral health instructions before the local anaesthetic was administered.

1. Stainless steel crowns: A flame-shaped diamond was used to reduce the occlusal surface uniformly by approximately 1.5 mm. A long and tapered diamond bur was used for proximal reduction to allow the probe to pass through the contact area. The suitable size of the crown was selected according to the mesiodistal dimension of the prepared tooth. Before cementation, a trial fit was conducted, as crowns should not extend more than 1 mm subgingivally. A correctly fitting crown should snap into place at try-in [28].
2. Zirconia and hybrid ceramic crowns: The occlusal surface was reduced by 1–2 mm via a flame bur, followed by opening of the interproximal areas. The crown dimensions were reduced by 0.5–1.25 mm via a tapered diamond bur, making the contour of the prepared tooth consistent with the natural contour. A pointed tapered diamond bur was used to make a 1–2 mm subgingival feather-edge preparation. Each crown had to have a passive fit that would prevent the formation of microcracks in its structure.
  • The appropriate size of the crown was selected and tested for appropriate fit before the final cementation. The prepared tooth was cleansed from blood, saliva and preparation remnants to make it ready for cementation [15]. The crown was subsequently cleaned, filled with cement and applied to the tooth until it was fully seated. A probe and floss were used to remove any excess cement. Resin-modified glass ionomer cement (GC FUJI I GOLD LABEL POWDER & LIQUID CEMENT) was used for the cementation of all the crowns. All the clinical procedures from crown preparation until crown cementation were performed by the same operator, and all the crowns were placed via a standardized crown placement protocol.
  • Bite evaluation: After crown placement, the bite must be assessed for any occlusal discrepancies or high points, as these could influence wear patterns and outcomes.

### Evaluation

**Fig. 4:**
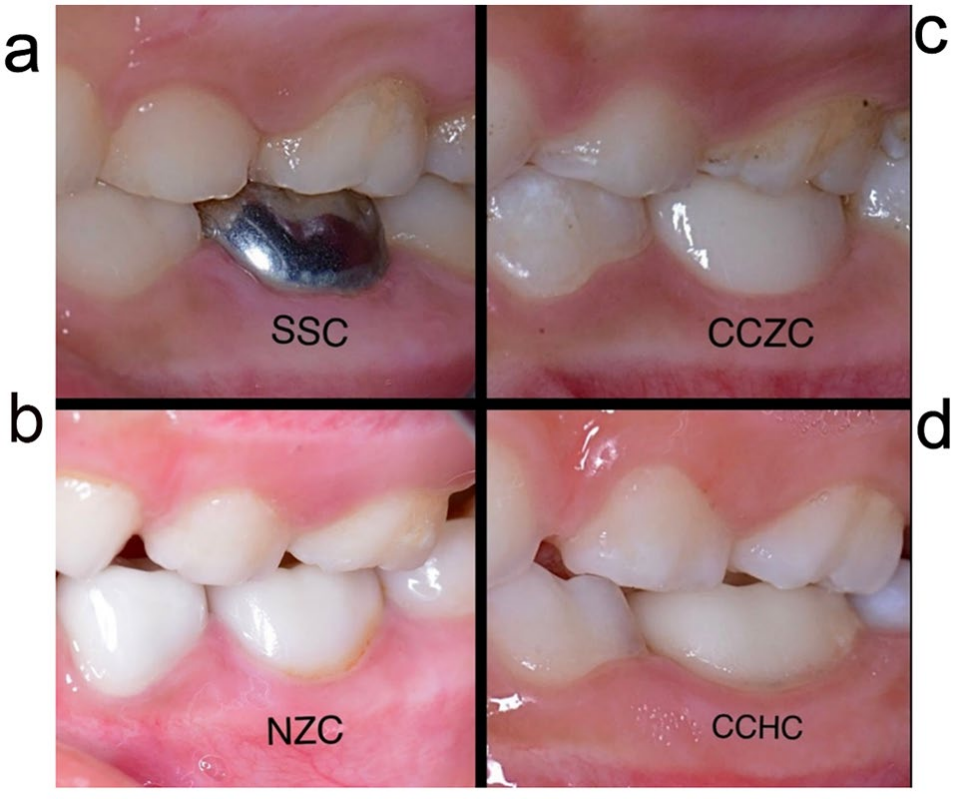
Occlusal relationships of 4 different crowns with the opposing upper natural teeth. (**A**) Occlusal relationship of the SSC with the opposing upper natural teeth. (**B**) Occlusal relationship of the NZC with the opposing upper natural teeth. (**C**) Occlusal relationship of the CCZC with the opposing upper natural teeth. (**D**) Occlusal relationship of the CCHC with the opposing upper natural teeth

**Fig. 5:**
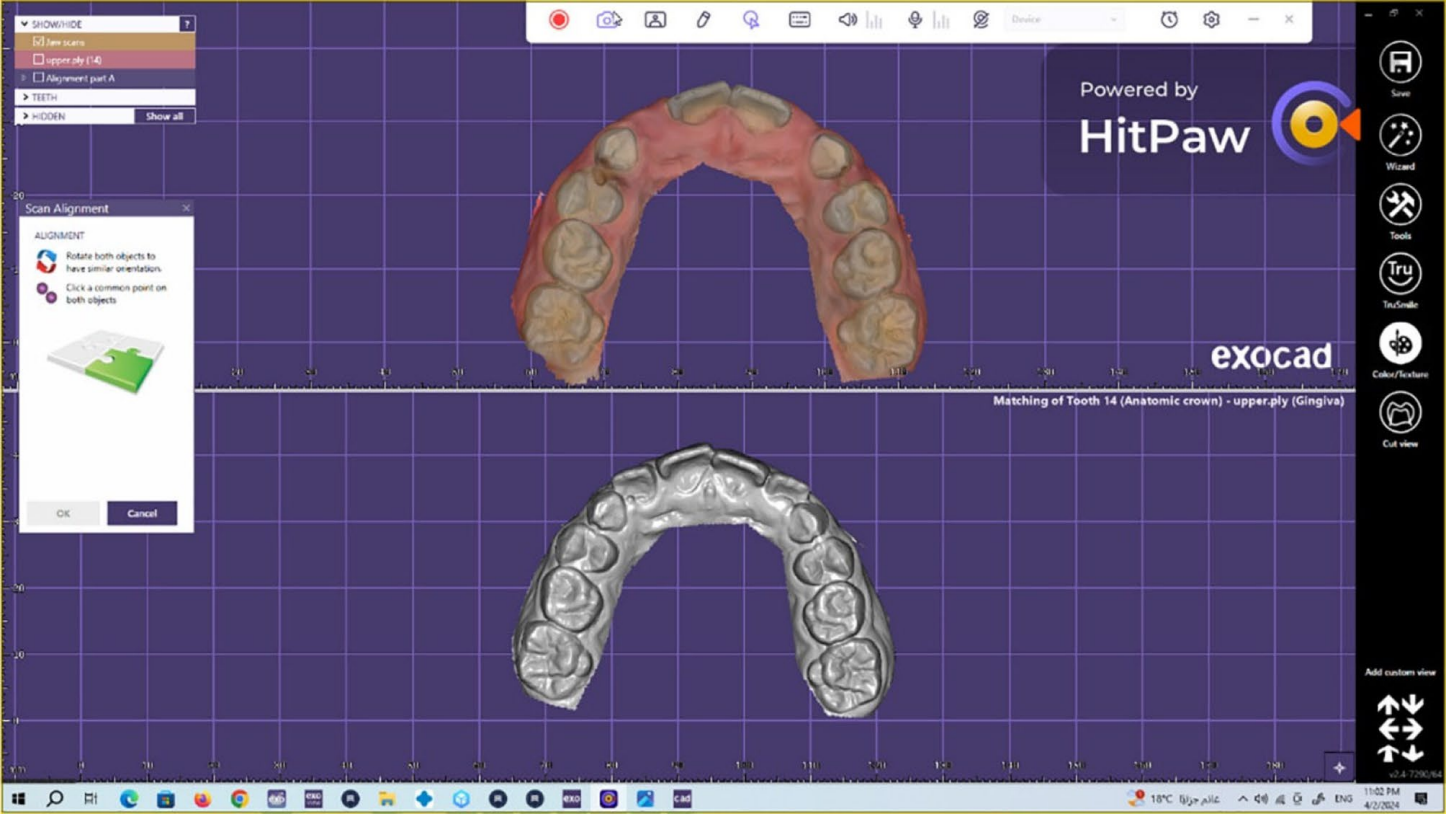
Method of measuring the wear of the natural enamel of the antagonistic tooth by using the Exocad program

**Fig. 6:**
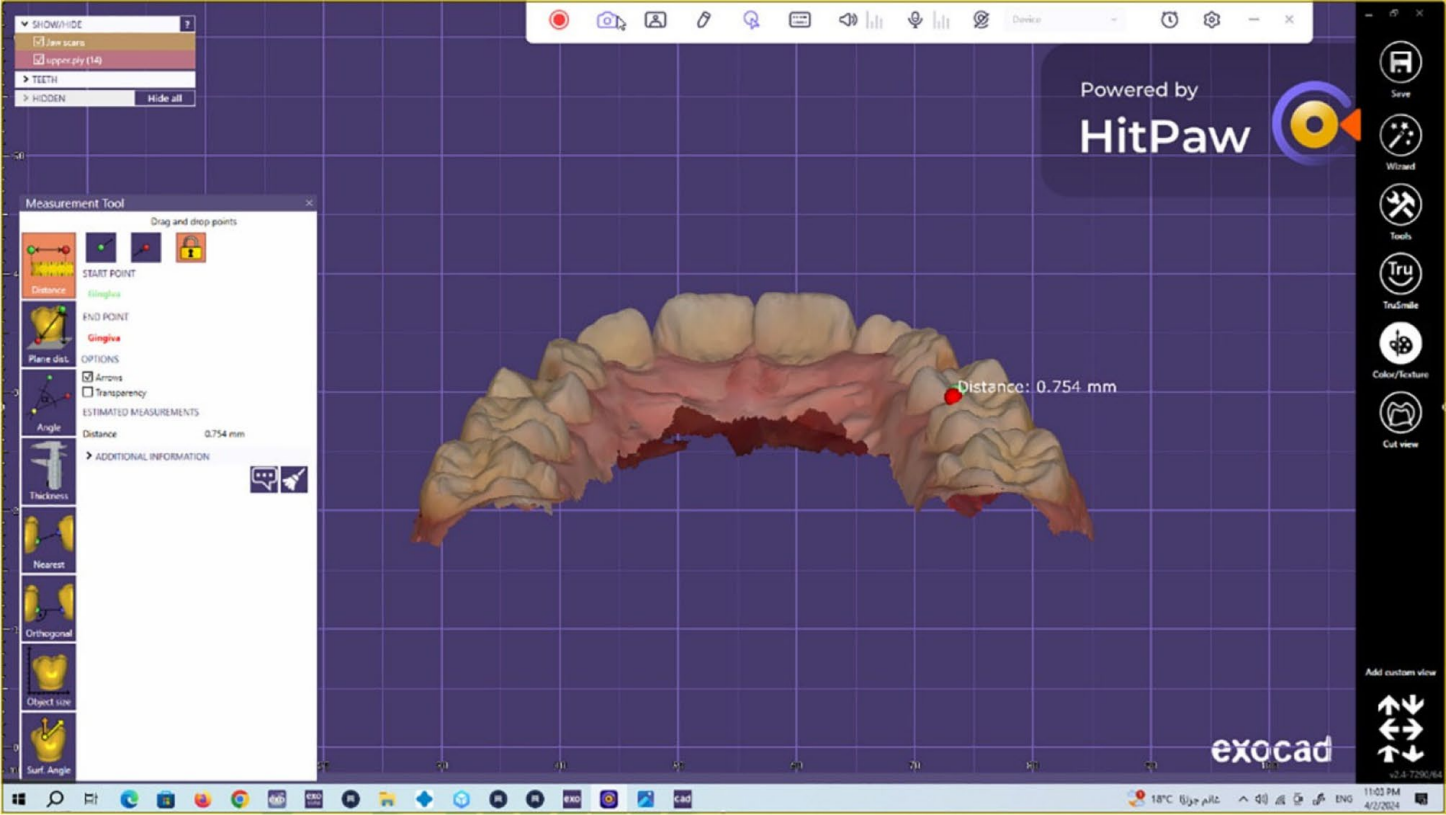
Method of measuring the wear of the natural enamel of the antagonistic tooth by using the Exocad program

To assess the wear of the natural enamel of antagonistic primary molars over 1 year [21, 29], the upper arch was scanned via an intraoral scanner (NEOSCAN™) immediately (baseline), 6 months, and 1 year after cementation. The software of the scanner transformed the scan data into a standard tessellation language (STL) file, thereby producing a 3D image of the jaw. Both 3D images were placed in the same position by matching landmark points on both images (superimposition). The Exocad software (Rijeka 3.1. Darmstadt, Germany) was used to measure the difference in the height of the cusp tip of the mesiopalatal cusp of the primary maxillary second molars in millimetres to calculate the amount of linear wear (quantitative analysis). The evaluation was performed with the help of a dental technician (Figs. 4, 5, 6).

### Statistical analysis

The data were analysed via the Statistical Package for Social Sciences (SPSS) version 25. The Shapiro‒Wilk test was used to test quantitative data for normality, which were then reported as standard deviations and means for normally distributed data. Comparisons between different groups for normally distributed data were performed via one-way ANOVA. Two-way ANOVA was used to study the combined effect of 2 independent factors (random effect of group and fixed effect of time) on the dependent continuous outcome (wear), with an estimation of R2.

## Results

**Table 1:** One-way ANOVA test for comparison of wear between the different studied groups at 6 months

	SCC	NZC	CCZC	CCHC	Test of significance
Wear at 6 months	0.0993 ± 0.044^a^	0.391 ± 0.038^b^	0.594 ± 0.063^c^	0.212 ± 0.055^d^	F = 176.4 *P* < 0.001*

Different superscripted letters denote statistically significant difference between studied groups

**Fig. 7:**
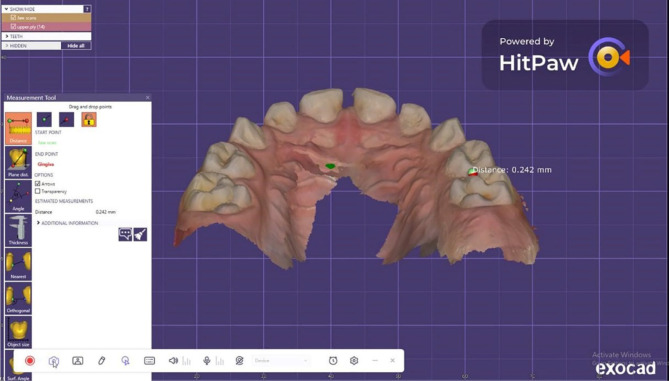
Linear wear of the natural enamel of the tooth antagonistic to the CAD/CAM hybrid crown (CCHC) as measured using the Exocad program at the 6-month follow-up

**Fig. 8:**
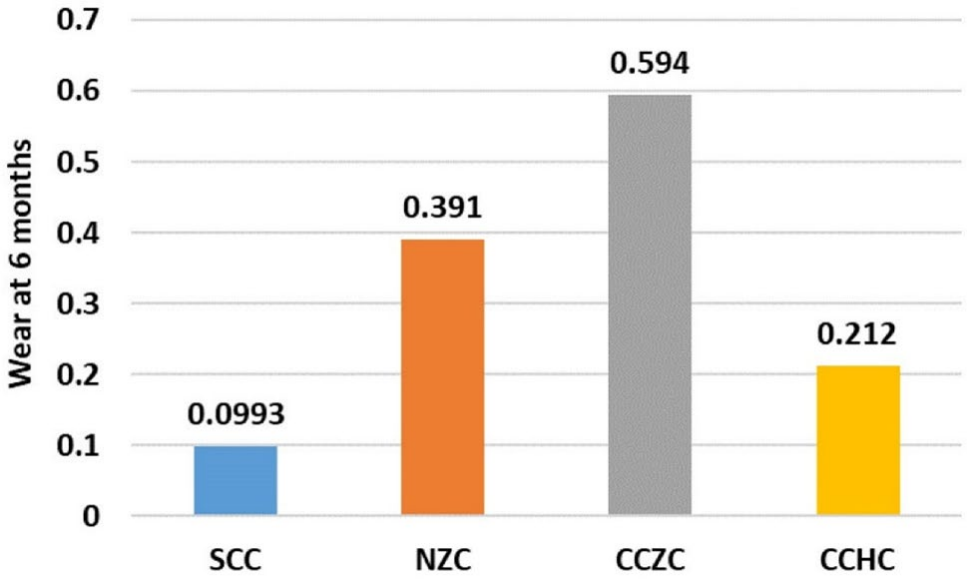
Comparison of wear at 6 months between the different studied groups

As shown in Table 1, there was a statistically significant difference in wear at 6 months between the different studied groups (F = 176.4, *P* < 0.001), with the highest mean wear detected for the CCZC group, followed by the NZC group and CCHC group, whereas the lowest mean wear was detected for the SSC group (0.594 ± 0.06, 0.391 ± 0.04, 0.212 ± 0.05 and 0.099 ± 0.04, respectively). Furthermore, pairwise comparisons between each of the studied groups revealed statistically significant differences according to the post hoc Tukey test (all *P* < 0.001) (Figs. 7, 8).

**Table 2:** One-way ANOVA test for comparison of wear between the different studied groups at 12 months

	SCC	NZC	CCZC	CCHC	Test of significance
Wear at 12 months	0.194 ± 0.04^a^	0.551 ± 0.069^b^	0.768 ± 0.057^c^	0.347 ± 0.065^d^	F = 172.66 *P* < 0.001*

Different superscripted letters denote statistically significant difference between studied groups

**Fig. 9:**
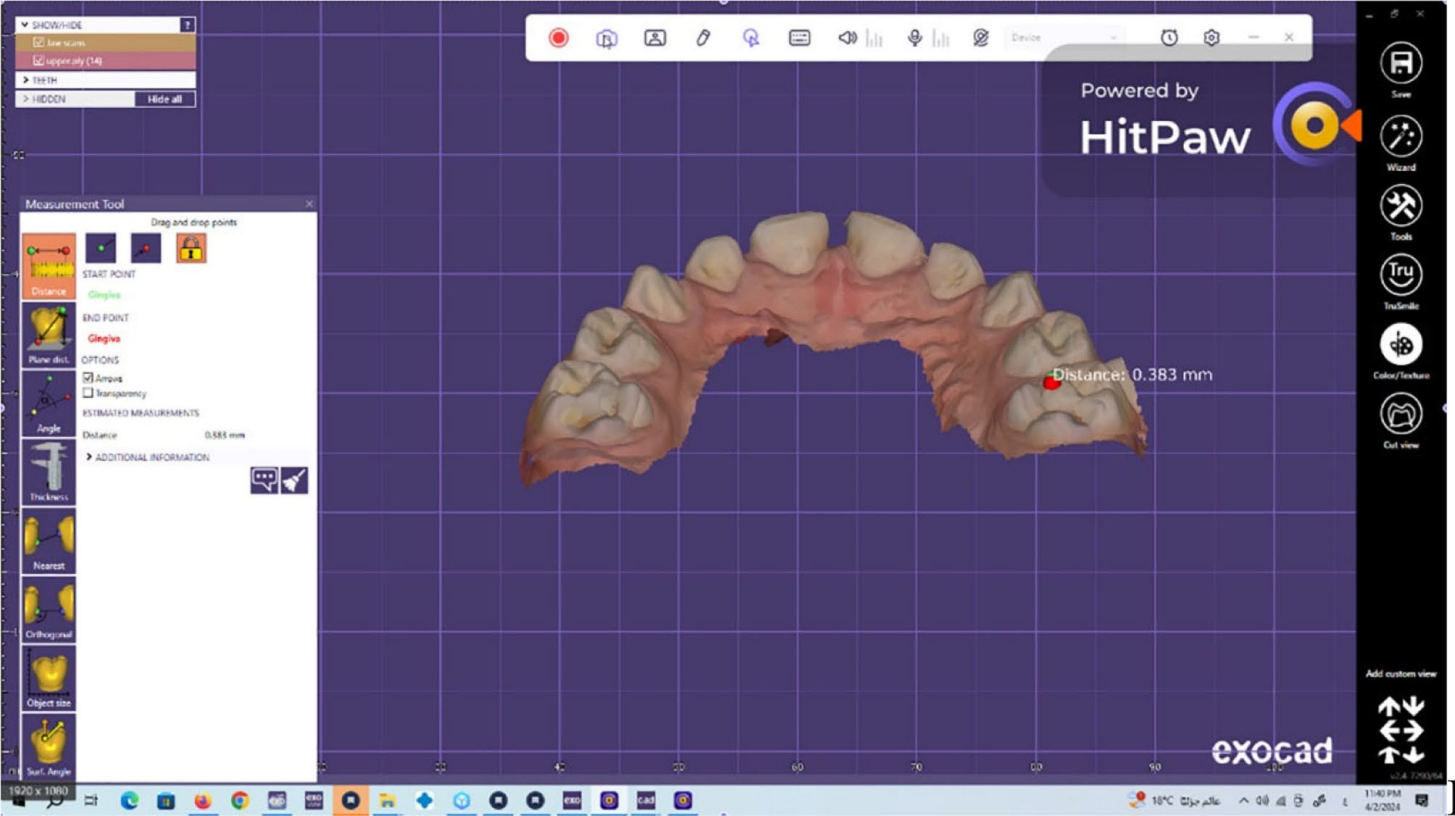
Linear wear of the natural enamel of the tooth antagonistic to the CAD/CAM hybrid crown (CCHC) as measured using the Exocad program at the 12-month follow-up

**Fig. 10:**
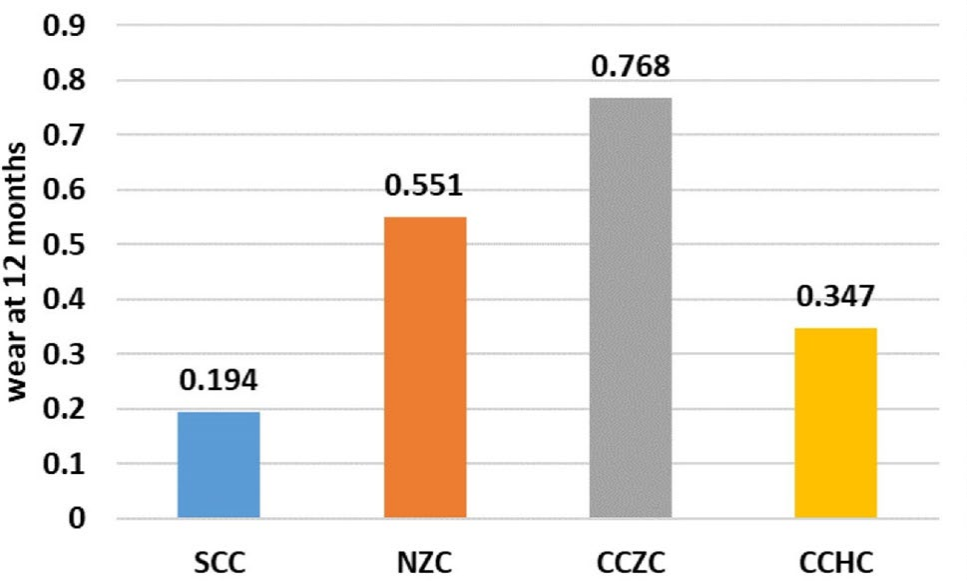
Comparison of wear at 12 months between the different studied groups

As shown in Table 2, there was a statistically significant difference in wear at 12 months between the different studied groups (F = 172.66, *P* < 0.001), with the highest mean wear detected for the CCZC group, followed by the NZC group and then the CCHC group, and with the lowest wear detected for the SSC group (0.768 ± 0.057^,^ 0.551 ± 0.069^,^ 0.347 ± 0.065 and 0.194 ± 0.04, respectively). Pairwise comparisons between each of the studied groups revealed statistically significant differences according to the post hoc Tukey test (all *P* < 0.001) (Figs. 9, 10).

**Table 3:** Comparison of changes in wear between the 6- and 12-month follow-ups among the different groups

	at 6 months	at 12 months	test of significance	% Of change
SCC	0.0993 ± 0.044	0.194 ± 0.04	t = 8.13 *p* < 0.001*	95.9%
NZC	0.391 ± 0.038	0.551 ± 0.07	t = 9.24 *p* < 0.001*	40.7%
CCZC	0.594 ± 0.064	0.768 ± 0.057	t = 6.89 *p* < 0.001*	29.5%
CCHC	0.212 ± 0.06	0.347 ± 0.065	t = 10.58 *p* < 0.001*	61.1%

t: Paired t test, *statistically significant

**Fig. 11:**
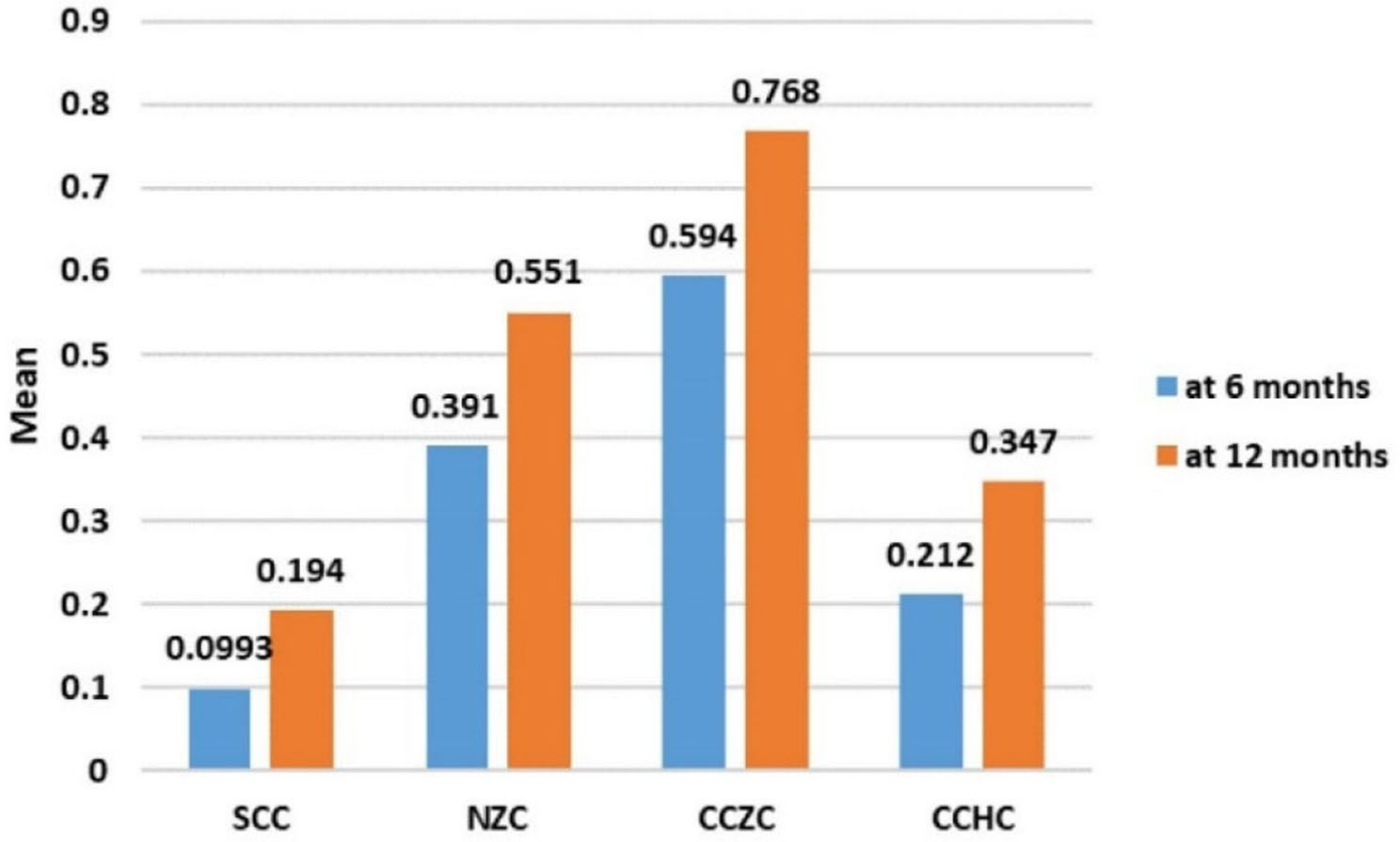
Comparison of wear at 6 and 12 months between the studied groups

There was a statistically significant increase in the mean wear after 12 months compared with 6 months for each of the studied groups (Table 3). The highest percentage increase in wear was detected for the SCC group, followed by the CCHC, NZC and CCZC groups (95.9%, 61.1%, 40.7% and 29.5%, respectively). There was a statistically significant difference in the percentage change between the SSC group and the NZC group (*P* = 0.007) and between the SSC group and the CCZC group (*P* = 0.002) (Figure 11).

**Table 4:** Two-way ANOVA test for the prediction of wear by the combined effect of changes in time and group

Source	Type III Sum of Squares	Df	Mean Square	F	*P* value
Corrected Model	3.669^a^	7	0.524	167.552	0.001*
Intercept	12.447	1	12.447	3979.068	0.001*
Time	0.398	1	0.398	127.379	0.001*
Group	3.252	3	1.084	346.544	0.001*
Time * group	0.018	3	0.006	1.951	0.129
Error	0.225	72	0.003		
Total	16.341	80			
Corrected Total	3.894	79			

a. R Squared = 0.942 (Adjusted R Squared = 0.937)

Table 4 Two-way ANOVA was used to assess the effects of changes in group and time of assessment on continuous outcomes (wear). The results showed that the time factor alone and the group factor alone had statistically significant effects on wear change, whereas the combined change in time and group had no statistically significant effect on wear change (F = 1.95, *P* = 0.129), with 93.7% of wear changes affected by these 2 factors.

## Discussion

Tooth wear is a complex multifactorial phenomenon that can cause hypersensitivity of dentin, functional and aesthetic problems, and loss of the vertical dimension, which in turn can cause temporomandibular disorders, overeruption of opposing teeth and traumatic occlusion [30]. Compared with natural teeth, dental materials can affect the wear rates of the opposing natural teeth if they have different wear properties [31]. Therefore, the aim of this study was to evaluate the antagonistic enamel wear of primary molars opposed to four different crown materials. The results revealed that CAD/CAM zirconia crowns were the most abrasive to their antagonists.

The wear of the natural tooth was evaluated via an intraoral scanner, as this device provides direct digitalization by scanning the oral cavity with a camera, which can significantly decrease errors related to impression taking and the fabrication of models [32]. Because the mesiopalatal cusp of primary maxillary second molars is the functional cusp, it was chosen to calculate the amount of linear wear of the enamel antagonistic to the four types of crowns [25].

The 3D scans made at 6- and 12-month intervals were superimposed via Exocad software, as the growth that occurred at 6 to 12 months was not significant enough to prevent the superimposition of 2 scans. Laowansiri et al. (2013) reported that maxillary and anterior cranial base growth rates are greatest during the first year and then decelerate over the next 4 years. Additionally, they reported that the overall growth changes during the first 5 postnatal years, especially during the first 2–3 years, are generally greater than the changes between 5 and 16 years [33].

This study reported a statistically significant difference between each pair, with the highest mean wear detected for the CCZC group, followed by the NZC group and then CCHC group, and with the least wear detected for the SSC group. The zirconia crowns caused the most aggressive wear of the antagonistic natural tooth. This finding is in accordance with the studies of Agrawal et al. [34] and Aly et al. [22] who concluded that stainless steel crowns resulted in less wear of the opposing natural tooth than did zirconia crowns. Additionally, Peng et al. 2023 [27] reported that zirconia crowns were the most abrasive to their antagonists, and Shan-Li Pei and Min-Huey Chen 2023 [35] concluded that zirconia crowns caused wearing and chipping of the opposite tooth. In contrast, Bolaca A and Erdoğan Y 2019 [25] concluded that zirconia caused less wear of the opposing tooth than did resin nanoceramics.

Moreover, Prabhu et al. reported that, compared with custom-made CAD/CAM zirconia crowns, SSCs yielded improved results in terms of opposing tooth wear in primary molars. Mohn et al. [26] found that zirconia crowns not only demonstrated wear resistance but also caused high wear of the antagonistic tooth. The increased wear rate imposed by zirconia was explained by the mechanical mismatch between the human enamel and the zirconia crown. Human enamel has a flexural strength of 280 GPa, hardness of 3.2 GPa and modulus of elasticity of 94 GPa; in contrast, zirconia crowns have a flexural strength > 1,000 MPa, hardness of 10 GPa and elastic modulus of 210 GPa, which are all greater than those of natural enamel [22].

In this study, the CAD/CAM hybrid materials resulted in less wear of the opposing natural enamel than did the zirconia crowns, as the CAD/CAM resin-matrix-based materials have a higher modulus of resilience than ceramics do. Under force, these materials undergo elastic deformation by distributing the stresses, and consequently, they tend to be more flexible and less brittle than ceramics [36]. Previous in vitro studies have shown that resin nanoceramics cause less antagonist enamel wear than glass ceramics do [37]. Additionally, Mohn et al. [26] reported that hybrid materials seem to be antagonist-friendly. Furthermore, Santos et al. (2018) [24] reported that Vita Enamic^®^ had low wear resistance.

In the present study, NuSmile zirconia resulted in less wear of the antagonistic enamel than did CAD/CAM zirconia. This can be explained by the manufacturing method of NuSmile prefabricated zirconia crowns, which uses an injection moulding technique and hand smoothing followed by mechanical polishing, which lowers the surface roughness (Ra 2.8) and increases the mean gloss (Ga 42.7) [38]. The above features result in less wear of the opposing tooth.

A comparison of the wear results at 6 and 12 months revealed that the highest percentage increase in wear was detected for the SCC group, followed by the CCHC group, whereas the lowest percentage increase in wear was detected for zirconia crowns. This can be explained by the surface roughness of the crown being positively correlated with the rate of antagonistic tooth wear [39]. The surface of SSC is prone to scratches with time, which increase the surface roughness [40]. Santos et al. (2018) [24] reported that Vita Enamic^®^ results in material loss from its surface due to the lack of adhesion between the ceramic particles and the polymeric matrix, which increases surface roughness. On the other hand, zirconia crowns have superior surface hardness, which makes them resistant to scratches [41].

A limitation of this study is the unavailability of prefabricated CAD/CAM crowns on the market, as they require special laboratory work. In addition, the extent of enamel wear over short follow-up periods may differ from long-term patterns; consequently, more clinical studies are needed to evaluate enamel wear over the long term.

## Conclusion

Compared with stainless steel crowns, aesthetic crowns induce more wear of the antagonistic primary molar enamel. CAD/CAM zirconia crowns induce the greatest amount of wear, followed by NuSmile zirconia crowns. The CAD/CAM hybrid crown is an aesthetic tooth-coloured crown that causes less wear of the opposing enamel than zirconia crowns do.

### Clinical significance

The use of CAD/CAM hybrid crowns in patients with bruxism is recommended when aesthetics is a prime concern

## Electronic supplementary material

Below is the link to the electronic supplementary material.

Supplementary Material 1

Supplementary Material 2

## Data Availability

Availability of data and materials Upon reasonable request, all of the datasets utilized and analyzed in this study are accessible from the corresponding author.

## Abbreviations

SSC: Stainless Steel Crown
CAD/CAM: Computer-Aided Design/Computer-Aided Manufacturing
CCZC: CAD/CAM Zirconia Crown
CCHC: CAD/CAM Hybrid Crown
STL: Standard Tessellation Language
